# Validation of Revised International Creutzfeldt-Jakob Disease Surveillance Network Diagnostic Criteria for Sporadic Creutzfeldt-Jakob Disease

**DOI:** 10.1001/jamanetworkopen.2021.46319

**Published:** 2022-01-31

**Authors:** Neil Watson, Peter Hermann, Anna Ladogana, Angeline Denouel, Simone Baiardi, Elisa Colaizzo, Giorgio Giaccone, Markus Glatzel, Alison J. E. Green, Stéphane Haïk, Daniele Imperiale, Janet MacKenzie, Fabio Moda, Colin Smith, David Summers, Dorina Tiple, Luana Vaianella, Gianluigi Zanusso, Maurizio Pocchiari, Inga Zerr, Piero Parchi, Jean-Philippe Brandel, Suvankar Pal

**Affiliations:** 1 National CJD Research & Surveillance Unit, Centre for Clinical Brain Sciences, University of Edinburgh, Edinburgh, United Kingdom; 2 National Reference Centre for TSE, Department of Neurology, University Medical Centre Göttingen, Göttingen, Germany; 3 Registry of Creutzfeldt-Jakob Disease, Department of Neuroscience, Istituto Superiore di Sanità, Rome, Italy; 4 Cellule Nationale de référence des MCJ, Groupe Hospitalier Pitié-Salpêtrière, Paris Cedex 13, France; 5 IRCCS Istituto delle Scienze Neurologiche di Bologna, Programma Neuropatologia delle Malattie Neurodegenerative, Bologna, Italy; 6 Department of Experimental, Diagnostic and Specialty Medicine, University of Bologna, Bologna, Italy; 7 Neurology 5/Neuropathology Unit, Fondazione IRCCS Istituto Neurologico C. Besta, Milan, Italy; 8 Institute of Neuropathology, University Medical Centre Hamburg-Eppendorf, Hamburg, Germany; 9 SC Neurologia 1, Ospedale Maria Vittoria, Torino, Italy; 10 Department of Neurosciences, Biomedicine, and Movement Sciences, University of Verona, Policlinico G.B. Rossi, Verona, Italy

## Abstract

**Importance:**

Sporadic Creutzfeldt-Jakob disease (sCJD) is a rapidly lethal disease. Rapid, accurate diagnosis is imperative for epidemiological surveillance and public health activities to exclude treatable differentials and facilitate supportive care. In 2017, the International CJD Surveillance Network diagnostic criteria were revised to incorporate cortical ribboning on magnetic resonance imaging and the real-time quaking-induced conversion (RT-QuIC) assay, developments that require multicenter evaluation.

**Objective:**

To evaluate the accuracy of revised diagnostic criteria through the retrospective diagnosis of autopsy-confirmed cases (referred to as *in-life diagnosis*).

**Design, Setting, and Participants:**

This diagnostic study used a 3-year clinicopathological series using all cases of autopsy-confirmed sCJD and a noncase group with alternative neuropathological diagnoses from national surveillance centers in the United Kingdom, France, Germany, and Italy. Data were collected from January 2017 to December 2019 and analyzed from January 2020 to November 2021.

**Main Outcomes and Measures:**

Sensitivity and specificity of revised diagnostic criteria and diagnostic investigations. Secondary analyses assessing sCJD subgroups by genotype, pathological classification, disease duration, and age.

**Results:**

A total of 501 sCJD cases and 146 noncases were included. Noncase diagnoses included neurodegenerative diseases, autoimmune encephalitis, and cerebral insults such as anoxia. Participants in the sCJD cases cohort were younger (mean [SD] age, 68.8 [9.8] years vs 72.8 [10.9] years; *P* < .001) and had longer median (IQR) disease duration (118 [74.8-222.3] days vs 85 [51.5-205.5] days; *P* = .002); sex ratios were equivalent (253 [50.5%] male cases vs 74 [50.7%] male noncases). Sensitivity of revised criteria in in-life diagnosis (450 of 488 [92.2%] diagnoses; 95% CI, 89.5%-94.4%) was increased compared with prior criteria (378 of 488 [77.5%] diagnoses; 95% CI, 73.5%-81.1%; *P* < .001), while specificity (101 of 125 [80.8%] diagnoses; 95% CI, 72.8%-87.3%) was unchanged (102 of 125 [81.6%] diagnoses; 95% CI, 73.7%-88.0%; *P* > .99). Among 223 cases and 52 noncases with the full panel of investigations performed, sensitivity of revised criteria (97.8%; 95% CI, 94.9%-99.3%) was increased compared with prior criteria (76.2%; 95% CI, 70.1%-81.7%; *P* < .001) while specificity was unchanged (67.3%; 95% CI, 52.9%-79.7% vs 69.2%; 95% CI, 54.9%-81.3%; *P* > .99). In 455 cases and 111 noncases, cortical ribboning was 67.9% sensitive (95% CI, 63.4%-72.2%) and 86.5% specific (95% CI, 78.7%-92.2%). In 274 cases and 77 noncases, RT-QuIC was 91.6% sensitive (95% CI, 87.7%-94.6%) and 100% specific (95% CI, 96.2%-100%). Investigation sensitivity varied with genetic and pathological features, disease duration, and age.

**Conclusions and Relevance:**

This diagnostic study demonstrated significantly improved sensitivity of revised sCJD diagnostic criteria with unaltered specificity. The revision has enhanced diagnostic accuracy for clinical care and surveillance.

## Key Points

**Question:**

How sensitive and specific are the 2017 revised International Creutzfeldt-Jakob disease Surveillance Network diagnostic criteria for the diagnosis of sporadic Creutzfeldt-Jakob disease?

**Findings:**

In this diagnostic study of 647 individuals conducted by a multinational team of surveillance experts, the revised diagnostic criteria, which incorporate cerebrospinal fluid real-time quaking-induced conversion assay and cortical ribboning on brain magnetic resonance imaging, were significantly more sensitive than previous criteria with no loss of specificity.

**Meaning:**

These results suggest that the revised diagnostic criteria achieve superior diagnostic sensitivity and maintain a high degree of specificity compared with previous criteria, enhancing diagnostic accuracy for clinical care and surveillance.

## Teaser

This diagnostic study assesses the accuracy of revised guidelines for the diagnosis of Creutzfeldt-Jakob disease using autopsy-confirmed results from surveillance centers in France, Germany, Italy, and the United Kingdom.

## Introduction

Sporadic Creutzfeldt-Jakob disease (sCJD) is a rapidly progressive, universally lethal and transmissible prion disease.^1^ Incidence has been increasing across multiple nations for several decades.^1,2,3^ Clinical features include rapidly progressive cognitive decline with associated motor features and myoclonus progressing to akinetic mutism.^4^ Median survival is 5 months.^5^ International surveillance systems monitor epidemiological trends and address attendant public health concerns.^1^

Diagnosis can be challenging. With advanced diagnostics and declining autopsy rates,^6^ most cases are diagnosed antemortem. Accurate diagnosis is essential to exclude potentially reversible conditions that can mimic sCJD,^7,8^ which can facilitate appropriate supportive care^9^ and prompt public health actions to reduce transmission,^10^ as well as support the recruitment to clinical trials.^11^

The diagnostic criteria used by the International CJD Surveillance Network^12^ have evolved with development of investigations, initially incorporating electroencephalography (EEG)^13,14^ and subsequently including measurements of cerebrospinal fluid (CSF) 14-3-3 protein.^15,16^ Subsequently, basal ganglia hyperintensities on magnetic resonance imaging (MRI) were incorporated.^16^ The criteria were revised in 2017^12^ to incorporate multifocal cortical signal changes (ie, ribboning) in brain MRI and the real-time quaking-induced conversion (RT-QuIC) assay (Box).^17^ Hermann et al^18^ have reported these criteria to be 97% sensitive and 99% specific, enhancing classification of cases if the criteria had been applied in life (referred to as *in-life diagnosis*) as probable sCJD, the classification used for epidemiological monitoring by many national surveillance systems. Findings from this single-center study suggested that enhanced diagnostic accuracy accounted for some of the reported rising disease incidence via improved case ascertainment. Hermann et al recommended multicenter evaluation, as their study was unable to evaluate important factors such as prion protein gene *PRNP* codon 129 (c129) genotype and disease-associated prion protein (PrP^Sc^) glycotype combinations.^4,18,19,20,21^

Box. **2017 International CJD Surveillance Network Diagnostic Criteria for Sporadic CJD**

**1. Sporadic CJD**

**1.1.** Definite
  Progressive neurological syndrome + confirmation (neuropathological/immunocytochemical/biochemical)
**1.2.** Probable
  **1.2.1.** I + 2 of II + typical electroencephalography,^a^ OR
  **1.2.2.** I + 2 of II + typical magnetic resonance imaging brain,^b^ OR
  **1.2.3.** I + 2 of II + positive 14-3-3, OR
  **1.2.4.** Progressive neurological syndrome + positive real-time quaking-induced conversion (cerebrospinal fluid or other tissue)
**1.3.** Possible
  I + 2 of II + duration <2 years

Abbreviation: CJD, Creutzfeldt-Jakob disease.

^a^
Generalized periodic complexes.

^b^
High signal in caudate/putamen on magnetic resonance imaging brain scan or ≥2 cortical regions on diffusion-weighted imaging or fluid-attenuated inversion recovery.

This multinational clinicopathological diagnostic study evaluated the revised diagnostic criteria. We hypothesized that revised criteria were more sensitive than prior criteria and contributed to increased case ascertainment. We predicted variable sensitivity of EEG, MRI, and 14-3-3 but not RT-QuIC across c129 genotypes.^16,20,22,23^ We anticipated lower sensitivity among patients with atypical disease duration.

## Methods

We investigated data from national surveillance units in the United Kingdom (UK), France, Germany, and Italy. Neuropathological confirmation of sCJD is the criterion standard method, against which we sought to validate the 2017 diagnostic criteria. All autopsy-confirmed individuals with sCJD who died between January 2017 and December 2019 were included. A noncase control group was established using individuals with suspected CJD excluded on neuropathological examination (ie, autopsy or biopsy) during the same period.

We extracted information regarding demographic and clinical features. Age was defined by the date of tissue acquisition (by either autopsy or biopsy). We did not extract data regarding ethnicity of individuals. Cases were stratified into short, typical, and long survival groups by duration in the first, second and third, and fourth quartiles, respectively, and by age into decades. We collated results of diagnostic investigations, c129 genotype, and PrP^Sc^ type where available. Neuropathological diagnoses in noncases were classified into groups, including neurodegenerative, vascular, inflammatory, infectious, cerebral insults (including anoxia or seizures), miscellaneous diagnoses, and nondiagnostic examinations (eTable 1 in the Supplement).

In-life status was classified by the revised diagnostic criteria (Box). Individuals not meeting criteria definitions were classified as unclear*.* This category included individuals with limited clinical features and no positive investigation results as well as individuals with inadequate clinical features and a positive investigation that was not RT-QuIC (for example, an individual with ataxia and a positive 14-3-3 assay).

Research related to CJD surveillance has been approved as essential for public health purposes in the UK by the South East Scotland Research Ethics Service, the French Commission Nationale de l’Informatique et des Libertés, the German Federal Ministry of Health and ethics committee of University Medical Centre Göttingen, and the ethics committee of the Istituto superior di Sanità. Informed consent was obtained from all individuals assessed by the National CJD Research and Surveillance Unit (or their relatives, when participants had impaired cognitive capacity). For German individuals, all legal representatives consented to the scientific use of data; for Italian individuals, patients or their relatives provided consent for research linked to surveillance data; for French surveillance, informed consent was not required because surveillance is considered an essential public health activity. We followed the Standards for Reporting Diagnostic Accuracy (STARD) reporting guideline.

### Statistical Analysis

Sensitivity and specificity were calculated for individual investigations and for probable diagnosis by prior and revised criteria; denominators for each measure were the numbers of individuals with available investigation results that had sufficient information to allow classification by a given criteria. Sensitivity was defined as the percentage of cases with positive outcomes on individual investigations and for overall criteria classification as probable sCJD. Specificity was defined as the percentage of noncases with negative outcomes, both for individual investigations and for criteria (appropriate classification as outcome other than probable). Positive investigation outcomes were defined as per criteria. An investigation was classified positive if it had been positive at any stage during the individual’s assessment. Uninterpretable MRI and EEG sequences degraded heavily by artifact were excluded, as were CSF assays untestable for technical reasons. Weak-positive, indeterminate, or equivocal CSF results were treated as negative.

We assessed diagnostic criteria in 2 ways. The first was a real-world series using cases with clinical information available that had undergone any investigation. In the second, we performed an analysis restricted to cases with all investigations performed. For age comparisons, Student independent samples *t* tests and analysis of variance (ANOVA) were performed. Duration comparison was performed using the Mann-Whitney U test and Kruskal-Wallis test (with post hoc analysis between *PRNP* c129 groups using Dunn tests with Bonferroni correction factors). Categorical variables were assessed using χ^2^ or Fisher exact tests. McNemar tests were used to assess changes in diagnostic criteria performance. Statistical significance was set at a 2-sided *P* < .05. Analysis was performed using SPSS Statistics version 24 (IBM Corp).

## Results

### Demographics and Case Classification

A total of 501 individuals with sCJD (cases) and 146 with alternative diagnoses (noncases) were included (Table 1). Sex ratios for cases and noncases were equivalent (sCJD, 253 [50.5%] men vs noncases, 74 [50.7%] men; *P* = .98). Individuals with sCJD were significantly younger than those in the noncase cohort (mean [SD] age, 68.8 [9.8] years vs 72.8 [10.9] years; *P* < .001). Median (IQR) disease duration was longer in sCJD cases than for noncases (118 days [74.8-222.3] days vs 85 [51.5-205.5] days; *P* = .002).

**Table 1.** Demographic and Clinical Features of Study Cohort

Characteristics	Individuals, No. (%)		*P* value
	Cases (n = 501)	Noncases (n = 146)	
Demographics			
Sex^a^			
Men	253 (50.5)	74 (50.7)	.98
Women	247 (49.3)	72 (49.3)	
Age, mean (SD), y^a^^,^^b^	68.8 (9.8)	72.8 (10.9)	<.001
Duration, median (IQR), d^a^	118 (74.75-222.25)	85 (51.5-205.5)	.002
Biopsy	0	7 (4.8)	
Autopsy	501 (100)	139 (95.2)	
Clinical features			
RPCD	479 (98.2)	112 (89.6)	<.001
Myoclonus	334 (68.4)	52 (41.6)	<.001
Cerebellar	360 (73.8)	11 (8.8)	<.001
Visual	235 (48.2)	11 (8.8)	<.001
Pyramidal	215 (44.1)	33 (26.4)	<.001
Extrapyramidal	247 (50.6)	43 (34.4)	.001
Akinetic mutism	217 (44.5)	30 (24.0)	<.001

Abbreviation: RPCD, rapidly progressive cognitive decline.

^a^ *P* value from χ^2^ test for sex, Student *t* test for age, Mann-Whitney U test for duration.

^b^ Age at autopsy or biopsy.

Clinical information was available in 488 cases (97.2%) and 125 noncases (85.6%). All cardinal diagnostic features were significantly more prevalent in cases (eg, cerebellar features: sCJD, 360 individuals [73.8%] vs noncases, 11 individuals [8.8%]; *P* < .001) (Table 1). The percentage of cases classified as probable (ie, sensitivity) was significantly higher with revised criteria (92.2%; 95% CI, 89.5%-94.4% vs 77.5%; 95% CI, 73.5%-81.1%; *P* < .001) (Table 2). Of 42 cases previously classified as possible, 17 (3.5% of cohort) were reclassified as probable because of cortical ribboning on MRI, 5 (1.0%) by RT-QuIC, and 10 (2.1%) by both (Table 3). Of 68 cases previously classified as unclear, 40 (8.2%) were reclassified as probable by RT-QuIC.

**Table 2.** Performance of Diagnostic Criteria and Individual Investigations

Variable	No. with positive result/total No. (sensitivity %) [95% CI]^a^	No. with negative result/total No. (specificity %) [95% CI]^a^
Diagnostic criteria for probable sCJD		
Any investigation		
Revised	450/488 (92.2) [89.5-94.4]	101/125 (80.8) [72.8-87.3]
Prior	378/488 (77.5) [73.5-81.1]	102/125 (81.6) [73.7-88.0]
All investigations		
Revised	218/223 (97.8) [94.9-99.3]	35/52 (67.3) [52.9-79.7]
Prior	170/223 (76.2) [70.1-81.7]	36/52 (69.2) [54.9-81.3]
Investigations		
EEG	207/448 (46.2) [41.5-50.9]	104/118 (88.1) [80.9-93.4]
MRI (all)	395/455 (86.8) [83.4-89.8]	91/111 (82.0) [73.6-88.6]
CR and BG	181/455 (39.8) [35.3-44.4]	107/111 (96.4) [91.0-99.0]
CR alone	128/455 (28.1) [24.0-32.5]	100/111 (90.1) [83.0-94.9]
BG alone	86/455 (18.9) [15.4-22.8]	106/111 (95.5) [89.8-98.5]
CR (any)	309/455 (67.9) [63.4-72.2]	96/111 (86.5) [78.7-92.2]
BG (any)	267/455 (58.7) [54.0-63.2]	102/111 (91.9) [85.2-96.2]
14-3-3	326/453 (72.0) [67.6-76.0]	56/123 (45.5) [36.5-54.8]
RT-QuIC	251/274 (91.6) [87.7-94.6]	77/77 (100.0) [96.2-100]

Abbreviations: BG, basal ganglia; CR, cortical ribboning; EEG, electroencephalography; MRI, magnetic resonance imaging; RT-QuIC, real-time quaking-induced conversion; sCJD, sporadic Creutzfeldt-Jakob disease.

^a^ Sensitivity defined as positive outcome/total for cases. Specificity defined as negative outcome/total for noncases. For criteria, the outcome is classification as probable sCJD.

**Table 3.** Classification by Diagnostic Criteria

Classification^a^	Diagnoses, No. (%) [95% CI]		Change, %
	Prior	Revised	
**Any investigation**			
Cases (n = 488)			
Probable	378 (77.5) [73.5-81.1]	450 (92.2) [89.5-94.4]	14.7
Possible	42 (8.6) [6.3-11.5]	10 (2.1) [1.0-3.7]	−6.5
Unclear	68 (13.9) [11.0-17.3]	28 (5.7) [3.9-8.2]	−8.2
Noncases (n = 125)			
Probable	23 (18.4) [12.0-26.3]	24 (19.2) [12.7-27.2]	0.8
Possible	26 (20.8) [14.1-29.0]	25 (20.0) [13.4-28.1]	−0.8
Unclear	76 (60.8) [51.7-69.4]	76 (60.8) [51.7-69.4]	0
**All investigations**			
Cases (n = 223)			
Probable	170 (76.2) [70.1-81.7]	218 (97.8) [94.9-99.3]	21.5
Possible	14 (6.3) [3.5-10.3]	1 (0.5) [0.0-2.5]	−5.8
Unclear	39 (17.5) [12.7-23.1]	4 (1.8) [0.5-4.5]	−15.7
Noncases (n = 52)			
Probable	16 (30.8) [18.7-45.1]	17 (32.7) [20.3-47.1]	1.9
Possible	11 (21.2) [11.1-34.7]	10 (19.2) [9.6-32.5]	−1.9
Unclear	25 (48.1) [34.0-62.4]	25 (48.1) [34.0-62.4]	0

Abbreviation: RT-QuIC, real-time quaking-induced conversion.

^a^ Definitions of probable and possible classification are outlined in the diagnostic criteria. Individuals were classified as unclear if they did not fulfill other definitions, ie, those with inadequate clinical features with or without a supportive positive investigation (other than RT-QuIC).

There was no significant difference in specificity for revised and previous criteria (80.8%; 95% CI, 72.8%-87.3% vs 81.6%; 95% CI, 73.7%-88.0%; *P* > .99) (Table 2). Neuropathological data were available in 16 (67%) noncases fulfilling criteria for probable sCJD; diagnoses were Alzheimer disease (AD) (7 individuals [44%]; 1 had additional dementia with Lewy body [DLB] pathology), anoxic injury (3 [16%]), CD8+ encephalitis (2 [13%]), cerebrovascular disease (1 [6%]), DLB (1 [6%]), cerebral abscess (1 [6%]) and influenza-associated necrotizing encephalopathy (1 [6%]).

A total of 223 (44.5%) cases and 52 (35.6%) noncases underwent the full panel of investigations. Sensitivity for a probable diagnosis significantly increased using revised criteria (97.8%; 95% CI, 94.9%-99.3% vs 76.2%; 95% CI, 70.1%-81.7%; *P* < .001), while there was no significant difference in specificity between revised and prior criteria (67.3%; 95% CI, 52.9%-79.7% vs 69.2%; 95% CI, 54.9%-81.3%; *P* > .99) (Table 2).

### Diagnostic Investigations

Of diagnostic investigations, 455 cases and 111 noncases underwent MRI; sensitivity was 86.8% (95% CI, 83.4%-89.8%) and specificity 82.0% (95% CI, 73.6%-88.6%) (Table 2). For cortical ribboning, sensitivity was 67.9% (95% CI, 63.4%-72.2%) and specificity 86.5% (95% CI, 78.7%-92.2%); of 15 noncases (13.5%) with cortical ribboning, autopsy results were available for 10 (67%); diagnoses were 5 individuals with AD (50.0%; 2 had dual pathology [1 with copresent DLB, 1 with tauopathy]; seizures were present in 3), and 1 individual (10%) apiece with autoimmune encephalitis, hepatic encephalopathy with seizures, antiphospholipid syndrome, nonspecific encephalopathy, and nondiagnostic autopsy (eTable 2 in the Supplement). Basal ganglia hyperintensity was 58.7% sensitive (95% CI, 54.0%-63.2%) and 91.9% specific (95% CI, 85.2%-96.2%) (Table 2). Of 9 (8%) noncases with basal ganglia hyperintensities, autopsy data were available in 5 (56%); diagnoses were 1 individual (20%) apiece with AD, dual AD and DLB, and DLB (2 autopsies [40%] were nondiagnostic). CSF RT-QuIC was the most sensitive (251 of 274 cases [91.6%; 95% CI, 87.7%-94.6%]) and specific (77 of 77 cases [100%; 95% CI, 96.2%-100%]) investigation; no positive results were identified in noncases.

### Subgroup Analysis

#### Codon 129 Genotype and PrP^Sc^ Type

Cases were grouped according to c129 genotype where possible (a total of 301 [60.0%] cases) yielding 3 groups: cases with methionine homozygosity (MM), cases heterozygous for methionine and valine (MV) and cases with valine homozygosity (VV) (Table 4). No differences were observed in the sensitivity of prior or revised diagnostic criteria between groups. MRI sensitivity did not differ between genotypes. Sensitivity of cortical ribboning on MRI was highest in MM (138 of 177 [78.0%; 95% CI, 71.1%-83.8%]), followed by MV (35 of 54 [64.8%; 95% CI, 50.6%-77.3%]) and VV (21 of 46 [45.7%; 95% CI, 30.9%-61.0%]) (*P* < .001) genotypes, while sensitivity of basal ganglia hyperintensity on MRI was highest in VV (44 of 46 [95.7%; 95% CI, 85.2%-99.5%]), followed by MV (40 of 54 [74.1%; 95% CI, 60.3%-85.0%]) and MM (88 of 147 [49.7%; 95% CI, 42.1%-57.3%]) genotypes (*P* < .001). Sensitivity of RT-QuIC did not vary between genotypes.

**Table 4.** Comparison Across sCJD c129 Polymorphism Subgroups

Characteristics	No. with positive result/total, No. (sensitivity %) [95% CI]			*P* value^b^
	MM (n = 196)^a^	MV (n = 57)^a^	VV (n = 48)^a^	
Demographics				
Sex, No. (%)				
Men	99 (50.5)	25 (43.9)	24 (50.0)	.34
Women	97 (49.5)	32 (56.1)	24 (50.0)	
Age, mean (SD), y	68.1 (10.3)	67.3 (9.4)	68.4 (8.4)	.67
Duration, median (IQR), d	112 (74-212)	376 (141-527)	170 (130-213)	.001^c^
Clinical features	165/190 (86.8) [81.2-91.3]	50/57 (87.7) [76.3-94.9)	44/47 (93.6) [82.5-98.7]	.44
Diagnostic criteria				
Revised	95/96 (99.0) [94.3-100]	27/27 (100.0) [87.2-100]	25/25 (100) [86.3-100]	.76
Prior	72/96 (75.0) [65.1-83.3]	20/27 (74.1) [53.7-88.9]	22/25 (88.0) [68.8-97.5]	.36
EEG	93/175 (53.1) [45.5-60.7]	20/50 (40.0) [26.4-54.8]	6/36 (16.7) [6.4-32.8]	<.001
MRI (all)	154/177 (87.0) [81.1-91.6]	49/54 (90.7) [79.7-96.9]	45/46 (97.8) [88.5-99.9]	.085
CR and BG	72/177 (40.7) [33.4-48.3]	26/54 (48.1) [34.3-62.2]	20/46 (43.5) [28.9-58.9]	.62
CR alone	66/177 (37.3) [30.2-44.9]	9/54 (16.7) [7.9-29.3]	1/46 (2.2) [0.1-11.5]	<.001
BG alone	16/177 (9.0) [5.3-14.3]	14/54 (25.9) [15.0-39.7]	24/46 (52.2) [37.0-67.1]	<.001
CR (any)	138/177 (78.0) [71.1 − 83.8]	35/54 (64.8) [50.6-77.3]	21/46 (45.7) [30.9-61.0]	<.001
BG (any)	88/177 (49.7) [42.1-57.3]	40/54 (74.1) [60.3-85.0]	44/46 (95.7) [85.2-99.5]	<.001
14-3-3	127/178 (71.4) [64.1-77.9]	23/50 (46) [31.8-60.7]	38/43 (88.4) [74.9-96.1]	<.001
RT-QuIC	110/117 (94) [88.0-97.6]	31/34 (91.2) [76.3-98.1]	27/29 (93.1) [77.2-99.2]	.08

Abbreviations: BG, basal ganglia hyperintensity; CR, cortical ribboning; EEG, electroencephalography; M, methionine; MRI, magnetic resonance imaging; RT-QuIC, real-time quaking-induced conversion assay; V, valine.

^a^ *P* values determined from χ^2^ tests for sex and sensitivity, ANOVA for age, Kruskal-Wallis tests for duration.

^b^ MM, MV, and VV refer to combinations of alleles.

^c^ Post-hoc analysis with Dunn test using Bonferroni correction factors demonstrated significant differences in disease duration between group MM and MV (*P* = .01), and MM and VV (*P* < .001). The difference between VV and MV groups was not significant (*P* = .15).

Cases were further grouped by c129 genotype and PrP^Sc^ type^4^ where possible (258 individuals [51.5%] in sCJD case cohort) (eTable 3 in the Supplement). Sensitivity of basal ganglia hyperintensities was highest in VV2 (35 of 37 [95%]) cases, while cortical ribboning was most sensitive in MM2 (11 of 14 [79%]) and MM1 (89 of 116 [77%]) cases. RT-QuIC showed sensitivity between 95% and 100% in all groups except MM2 (4 of 6 [67%]) and VV1 (negative in only case).

### Disease Duration and Age

The previous diagnostic criteria were most sensitive in cases with short (43 of 52 [82.7%; 95% CI, 69.7%-91.8%]) and typical (91 of 110 [82.7%; 95% CI, 74.4%-89.3%]) duration compared with those with prolonged duration (32 of 56 [57.1%; 95% CI, 43.2%-70.3%]) (*P* = .001) (eTable 4 in the Supplement). No group differences were observed with revised criteria. There were no differences in sensitivity of MRI, except in cases with isolated basal ganglia hyperintensity, which occurred most often in cases with prolonged duration (28 of 115 [24.4%; 95% CI, 16.8%-33.2%]), followed by typical (45 of 223 [20.2%; 95% CI, 15.1%-26.1%]) and short duration (11 of 106 [10.4%; 95% CI, 5.3%-17.8%]) (*P* = .02). RT-QuIC was most sensitive in cases with typical duration (126 of 131 [96.2%; 95% CI, 91.3%-98.7%]), followed by cases with prolonged (59 of 67 [88.1%; 95% CI, 77.8%-94.7%]) and short (59 of 68 [86.8%; 95% CI, 76.4%-93.8%]) duration (*P* = .03). Results of assessments of diagnostic criteria and investigation sensitivities between age groups are detailed in eTable 5 in the Supplement.

## Discussion

To our knowledge, this is the first large multinational clinicopathological evaluation of the 2017 International CJD Surveillance Network diagnostic criteria for sCJD, investigating a 3-year cohort of all neuropathologically confirmed sCJD cases from 4 national centers. Our study complements earlier observations,^18^ quantifying criteria performance and evaluating how criteria revisions may affect in-life case ascertainment. The revised criteria were 92.2% sensitive for probable sCJD diagnosis, rising to 97.8% when all investigations were performed, which resulted in a 21.5% improvement in antemortem case ascertainment compared with prior criteria. This improvement offers major utility for surveillance programs and clinicians.

We evaluated cases with full and partial investigation workup, reflecting the real-world limitations of surveillance. Reasons patients may not undergo all investigations include intolerance or contraindications to MRI or lumbar puncture^24^ and limitations to biomarker testing.^25^ Furthermore, after one positive investigation the diagnosis may be evident, thus making additional investigations unnecessary. Notably, the revised criteria demonstrated lower specificity than was reported (99%) in a previous single-center study^18^ in which noncases with evidence supporting alternative diagnoses were not classified as probable sCJD, regardless of positive sCJD-related investigations. Nonetheless, the reported sensitivity was similar (97%). We encourage full investigation where possible to maximize sensitivity, but emphasize the importance of considering alternative etiologies and investigating accordingly.^26^

Our study provides valuable information on investigation performance. MRI sensitivity (86.8%) was lower than typical figures (generally above 92%).^8,27^ MRI reporting by neuroradiologists with CJD expertise improves sensitivity.^28^ Methodological variations between centers may have reduced sensitivity, such as when reporting is conducted by radiologists without CJD experience. Our findings on specificity are valuable. 5 individuals with AD (including 2 with copathology; 1 with DLB and 1 with tauopathy) had cortical ribboning, 3 of whom had seizures, which can produce cortical DWI abnormalities.^29,30^ For the remaining 2, the explanation was uncertain. One study identified cortical MRI changes in 32% of nonprion dementias, with AD the most frequent etiology in the cohort.^31^ Other studies have not reported cortical ribboning in other neurodegenerative dementias.^8,27^ Five noncases with AD and 1 with DLB had basal ganglia abnormalities; these etiologies were not associated with basal ganglia DWI abnormalities in prior studies.^8,27^ Our findings support the need for future neuroimaging studies examining noncases. With expert reporting to maximize accuracy, future studies could assess the location of abnormalities, confirm diffusion restriction on apparent diffusion coefficient (ADC) mapping, and further examine features suggesting alternative diagnoses.

MRI abnormalities vary with CJD subtype.^32,33,34,35,36^ In our study, 78.0% of MM cases had cortical ribboning while 95.7% of VV cases had basal ganglia hyperintensities. Basal ganglia hyperintensities were most frequently encountered in VV2 (94.6%) and MV2 (77.3%) cases, results that are consistent with prior studies. MM2 (78.6%) and MM1 (76.7%) cases had the highest frequency of cortical ribboning. Studies have suggested predominant cortical ribboning in MM2 cases.^33,36^ The basis for sCJD-related DWI abnormalities remains uncertain,^37^ and different pathological features in distinct subtypes^4,38^ may factor into diffusion restriction patterns.

MRI sensitivity increases with disease progression.^39^ We observed no variation in sensitivity with total duration; however, we were unable to assess the impact of timing. Cases with a negative MRI might have developed diagnostic features on serial imaging. A recent study demonstrated high performance of MRI when criteria were expanded to include individuals with a single brain region affected.^40^ Such findings would be classified as negative according to the current diagnostic criteria. We recommend serial interval imaging of these individuals following the existing criteria; however, developing enhanced criteria permitting earlier diagnostic classification in such cases would be valuable.

RT-QuIC showed excellent (91.6%) sensitivity and 100% specificity, with no positive results in noncases. This outstanding specificity has been consistently reported.^25,41,42,43,44,45,46^ A small number of studies have reported positive RT-QuIC assays in individuals without prion disease but methodological doubts have been raised in relation to these.^25^ Autopsy-confirmed diagnoses included single cases of DLB,^47^ frontotemporal dementia with motor neuron disease,^48^ steroid-responsive encephalopathy,^49^ and AD with vascular dementia.^50^ The first individual had small amounts of PrP^Sc^, making dual pathology with subclinical sCJD possible.^25^ Other reported individuals did not undergo autopsy, and so CJD cannot be definitively excluded.^25^

RT-QuIC performance was independent of c129 genotype, which is in line with prior studies.^23,42,50^ The lower sensitivity in MM2 and VV1 cases is consistent with emerging evidence^25,46,47,50^. We included cases with co-occurring PrP^Sc^ types 1 and 2 (12.8%); these cases (up to 35% of sCJD^51^) are often excluded from studies,^35^ which oversimplifies findings and limits validity. Our study was underpowered to assess investigations in rare subtypes, and further work is necessary.

RT-QuIC was vastly more sensitive and specific than 14-3-3 assays. This finding has major value in challenging circumstances, such as for patients with atypical presentations, or in cases in which evidence suggests alternative diagnoses but a confirmed sCJD case would necessitate public health actions.

Previous criteria were less sensitive with prolonged duration, while no variation was seen with revised criteria, representing enhanced diagnostic capacity. The slight variation in RT-QuIC sensitivity with duration is of uncertain clinical significance. One study found no association between duration or timing of sampling with RT-QuIC sensitivity,^23^ while another found that shorter duration had some influence on seeding activity,^42^ and a 2020 study found longer survival and earlier sampling were associated with lower RT-QuIC sensitivity.^50^ We did not assess timing of investigations, which may have relevance in longer-surviving cases; further longitudinal work is necessary.

A major benefit of rapid and accurate in-life diagnosis is the potential for recruitment to therapeutic trials; challenges to recruitment include short survival, diagnostic latency,^52^ and rarity of CJD.^11,53^ Our results demonstrate that the diagnosis can be made with high confidence during life. A 2020 study^35^ explored noninvasive PrP^Sc^ subtyping using MRI to enable targeted trials. While our data support the consensus that MRI patterns correlate to strain subtypes, we did not evaluate the potential for MRI subtyping during life, and further work is necessary.

A total of 5 cases were not classified as probable sCJD by revised criteria when fully investigated; 4 were classified as unclear. The criteria do not classify individuals with insufficient clinical features, including those with a positive investigation (except RT-QuIC). This gap in criteria coverage poses challenges. In some individuals, sCJD likelihood is high, and a working diagnosis may be made, with follow-up demonstrating progression, allowing reclassification. sCJD is clinically heterogeneous,^4,51,54,55^ and some individuals may not satisfy criteria, for example cognitively spared individuals with ataxic onset. RT-QuIC has utility in such individuals, evidenced by the 40 cases reclassified from unclear to probable. Similarly, clinical features may evolve with disease progression. We recommend serial evaluation in individuals with unclear diagnoses. We propose the novel classification category “clinically limited sCJD” for individuals with limited clinical features and a supportive investigation (except RT-QuIC).

### Strengths and Limitations

Strengths of this study include its comprehensive multinational cohort of all cases of autopsy-confirmed sCJD from surveillance centers in a 3-year period. All cases and noncases had neuropathological confirmation or exclusion of sCJD, minimizing misclassification and allowing clinicopathological correlation, especially for positive investigations in noncases. We expanded on previous work by quantifying performance of diagnostic criteria in cases grouped by c129, PrP^Sc^, disease duration, and age.

This study has several limitations. Excluding individuals without neuropathological examination may have introduced bias. A minority of sCJD patients undergo autopsy in the modern era^6^; reasons include enhanced in-life diagnosis and declining autopsy service availability. Autopsied cases may overrepresent those for whom in-life diagnosis was not possible, reducing sensitivity. Noncases undergoing autopsy or biopsy are likewise a subgroup in surveillance; many do not undergo neuropathological examination and receive alternative diagnoses from clinical and investigation findings. Our sample may overrepresent noncases requiring autopsy, such as those with rapidly lethal illnesses or false positive investigations, which would lead to decreased specificity. Differences in methodology between centers may have been relevant, including a high proportion of German noncases with positive 14-3-3 assay.^18^ Availability of RT-QuIC and access to CJD specialist MRI reporting was not equivalent in all nations. Lastly, although our study explores internationally applied diagnostic criteria, it was centered in 4 large European nations. Additional analysis in other regions would be useful.

## Conclusions

In this study, we demonstrated the excellent performance of the revised CJD International Surveillance Network diagnostic criteria. Our results showed that the new criteria greatly enhanced in-life case classification, with improvements among cases with clinically limited sCJD and with prolonged survival. Rapid, accurate in-life diagnosis enables effective supportive care, public health interventions, and clinical trial recruitment.

## Notes

Supplement. **eTable 1.** Diagnoses Among Non-cases (Where Reports Available) **eTable 2.** Neuropathological Diagnoses in Non-cases With Positive Investigations (Where Available) **eTable 3.** sCJD Subtypes Defined by Codon 129 Genotype and PrP^Sc^ Glycotype Combinations **eTable 4.** Comparison of CJD Subjects According to Disease Duration **eTable 5.** Criteria and Investigation Sensitivity, sCJD Cases Stratified by Age
