# Supplementary material for: Validation of Revised International Creutzfeldt-Jakob Disease Surveillance Network Diagnostic Criteria for Sporadic Creutzfeldt-Jakob Disease
Source: JAMA Netw Open. 2022 Jan 31;5(1):e2146319. doi: 10.1001/jamanetworkopen.2021.46319 (PMC8804913; doi:10.1001/jamanetworkopen.2021.46319)
Supplement: Supplement. — eTable 1. Diagnoses Among Non-cases (Where Reports Available) eTable 2. Neuropathological Diagnoses in Non-cases With Positive Investigations (Where Available) eTable 3. sCJD Subtypes Defined by Codon 129 Genotype and PrPSc Glycotype Combinations eTable 4. Comparison of CJD Subjects According to Disease Duration eTable 5. Criteria and Investigation Sensitivity, sCJD Cases Stratified by Age [file jamanetwopen-e2146319-s001.pdf]

Supplemental Online Content

Watson N, Hermann P, Ladogana A, et al. Validation of revised International Creutzfeldt-Jakob Disease Surveillance Network diagnostic criteria for sporadic Creutzfeldt-Jakob disease. *JAMA Netw Open.* 2022;5(1):e2146319.
doi:10.1001/jamanetworkopen.2021.46319

eTable 1. Diagnoses Among Non-cases (Where Reports Available)

eTable 2. Neuropathological Diagnoses in Non-cases With Positive Investigations (Where Available)

eTable 3. sCJD Subtypes Defined by Codon 129 Genotype and PrP Glycotype Combinations

eTable 4. Comparison of CJD Subjects According to Disease Duration

eTable 5. Criteria and Investigation Sensitivity, sCJD Cases Stratified by Age

This supplemental material has been provided by the authors to give readers additional information about their work.

© 2022 Watson N et al. *JAMA Network Open.*

eTable 1. Diagnoses among non-cases (where reports available)

**Neurodegenerative**	**Vascular**	**Cerebral insult**	**Inflammation**	**Infection**	**Miscellaneous**	**Non-diagnostic examination**
Total (n=41)	Total (n=16)	Total (n=15)	Total (n=8)	Total (n=2)	Total (n=7)	Total (n=8)
AD (n=21)	Cerebrovascular disease (n=12)	Anoxia (n=8)	CD8+ encephalitis (n=3)	Abscess (n=1)	Metastatic RCC (n=1)	
Dual pathology (n=12)	Vasculitis (n=2)	Metabolic (n=2)	AIE (n=2)	Toxoplasmosis (n=1)	Fahr disease (n=1)	
AD + DLB (n=10)	APS (n=1)	Seizures (n=1)	Influenza-associated ANE (n=1)		Leukoencephalopathy (n=1)	
AD + tau (n=1)	CAA (n=1)	Anoxia + seizures (n=1)	Behcet's (n=1)		Gliosis (n=1)	
AD + MSA (n=1)		HE + seizures (n=1)	Inflammation NOS (n=1)		Encephalopathy NOS (n=1)	
DLB (n=3)		Hypoglycaemia (n=1)			Other, NOS (n=2)	
TDP43 (n=2)		Subcortical necrosis (n=1)				
Tauopathy (n=2)						
CBD (n=1)						

Abbreviations. AD, Alzheimer's. AIE, autoimmune encephalitis. ANE, acute necrotizing encephalitis. APS, antiphospholipid syndrome. CAA, cerebral amyloid angiopathy. CBD, corticobasal degeneration. DLB, dementia with Lewy Bodies. HE, hepatic encephalopathy. MSA, multiple systems atrophy. NOS, not otherwise specified. RCC, renal cell carcinoma

© 2022 Watson N et al. *JAMA Network Open.*

eTable 2. Neuropathological diagnoses in non-cases with positive investigations (where available)			
**Cortical ribboning**	**Basal ganglia hyperintensity**	**14-3-3**	**EEG**
Total (n=10)	Total (n=5)	Total (n=41)	Total (n=7)
AD (n=3)	AD + DLB (n=1)	AD (n=8)	AD (n=2)
AD + DLB (n=1)	AD + tau (n=1)	Anoxia (n=6)	APS (n=1)
AD + tau (n=1)	DLB (n=1)	Cerebrovascular disease (n=6)	Hypoglycaemia (n=1)
APS (n=1)	Non-diagnostic (n=2)	CD8+ encephalitis (n=3)	Anoxia + seizures (n=1)
HE + seizures (n=1)		AIE (n=2)	Tauopathy (n=1)
AIE (n=1)		AD + DLB (n=2)	Non-diagnostic (n=1)
Encephalopathy NOS (n=1)		TDP43 (n=1)	
Non-diagnostic (n=1)		HE + seizures (n=1)	
		Anoxia + seizures (n=1)	
		Seizure (n=1)	
		Hypoglycaemia (n=1)	
		Fahr disease (n=1)	
		Metastatic RCC (n=1)	
		Influenza-associated ANE (n=1)	
		Abscess (n=1)	
		Cerebral vasculitis (n=1)	
		Leukoencephalopathy (n=1)	
		Encephalopathy NOS (n=1)	
		Non-diagnostic (n=2)	
Abbreviations. AD, Alzheimer's. AIE, autoimmune encephalitis.ANE, acute necrotizing encephalitis. APS, antiphospholipid syndrome. CAA, cerebral amyloid angiopathy. CBD, corticobasal degeneration. DLB, dementia with Lewy Bodies. HE, hepatic encephalopathy. MSA, multiple systems atrophy. NOS, not otherwise specified. RCC, renal cell carcinoma			

eTable 3. sCJD subtypes defined by Codon 129 genotype and PrP glycotype combinations

	MM1	MM2	MM1+2	MV1	MV2	MV1+2	VV1	VV2	VV1+2
Demographics									
Total, n(%)	130 (50.4)	15 (5.8)	24 (9.3)	19 (7.4)	22 (8.5)	7 (2.7)	1 (0.4)	38 (14.7)	2 (0.8)
Sex, n (%)									
Male	70 (53.8)	5 (33.3)	8 (33.3)	9 (47.4)	8 (36.4)	2 (28.6)	1 (100.0)	18 (47.4)	1 (50.0)
Female	60 (46.2)	10 (66.7)	16 (66.7)	10 (52.6)	14 (63.6)	5 (71.4)	-	20 (52.6)	1 (50.0)
Age, years (SD)	68.4 (10.3)	64.0 (12.2)	69.6 (9.4)	73.3 (5.5)	68.0 (9.0)	59.2 (14.1)	60.0 (-)	68.7 (9.0)	70.5 (5.0)
Duration, days (IQR)	92.5 (67.8-150)	451 (307-661)	120 (88-223)	173 (65-374)	411.5 (341.5-668.25)	539 (309.5-649.5)	320 (-)	170 (128-203)	231 (-)
Investigation sensitivity (%)									
EEG	69/118 (58.5)	4/12 (33.3)	11/22 (50.0)	11/19 (57.9)	4/18 (22.2)	3/6 (50.0)	0/1 (0.0)	4/34 (11.8)	0/2 (0.0)
MRI (all)	102/116 (87.9)	12/14 (85.7)	20/24 (83.3)	14/17 (82.4)	21/22 (95.5)	7/7 (100.0)	1/1 (100.0)	36/37 (97.3)	2/2 (100.0)
CR & BG	53/116 (45.7)	3/14 (21.4)	8/24 (33.3)	10/17 (58.8)	7/22 (31.8)	6/7 (85.7)	1/1 (100.0)	15/37 (40.5)	1/2(50.0)
CR alone	36/116 (31.0)	8/14 (57.1)	10/24 (41.7)	2/17 (11.8)	4/22 (18.2)	1/7 (14.3)	0/1 (0.0)	1/37 (2.7)	0/2 (0.0)
BG alone	13/116 (11.2)	1/14 (7.1)	2/24 (8.3)	2/17 (11.8)	10/22 (45.5)	0/7 (0.0)	0/1 (0.0)	20/37 (54.1)	1/2 (50.0)
CR (any)	89/116 (76.7)	11/14 (78.6)	18/24 (75.0)	12/17 (70.6)	11/22 (50.0)	7/7 (100.0)	1/1 (100.0)	16/37 (43.2)	1/2 (50.0)
BG (any)	66/116 (56.9)	4/14 (28.6)	10/24 (41.7)	12/17 (70.6)	17/22 (77.3)	7/7 (85.7)	1/1 (100.0)	35/37 (94.6)	2/2 (100.0)
14-3-3	94/117 (80.3)	3/13 (23.1)	12/23 (52.2)	11/16 (68.8)	6/19 (31.6)	2/6 (33.3)	1/1 (100.0)	30/34 (88.2)	2/2 (100.0)
RT-QuIC	79/83 (95.2)	4/6 (66.7)	16/17 (94.1)	14/14 (100.0)	14/14 (100.0)	2/2 (100.0)	0/1 (0.0)	22/23 (95.7)	2/2 (100.0)

Age shown as mean, duration as median.

Abbreviations: BG, basal ganglia. CR, cortical ribboning. EEG, electroencephalography. IQR, interquartile range. MRI, magnetic resonance imaging. RT-QuIC, real-time quaking-induced conversion assay. SD, standard deviation.

© 2022 Watson N et al. *JAMA Network Open.*

**eTable 4. Comparison of CJD subjects according to disease duration**				
	**Disease duration (days)**			
	**Short (<75) (n=121)**	**Typical (75-222) (n=242)**	**Prolonged (>222) (n=121)**	**P value**
Sensitivity (%) [95% CI]				
Diagnostic criteria				
Clinical features	102/120 (85.0) [77.3-90.7]	213/239 (89.1) [84.5-92.8]	98/119 (82.4) [74.3-88.7]	.19
Probable sCJD				
Revised criteria	51/52 (98.1) [89.8-100]	109/110 (99.1) [95.0-100]	53/56 (94.6) [85.1-98.9]	.19
Prior criteria	43/52 (82.7) [69.7-91.8]	91/110 (82.7) [74.4-89.3]	32/56 (57.1) [43.2-70.3]	**.001**
Investigations				
EEG	68/109 (62.4) [52.6-71.5]	105/225 (46.7) [44.2-58.2]	28/105 (26.7) [18.5-36.2]	**<.001**
MRI	92/106 (86.8) [78.8-92.6]	190/223 (85.3) [79.9-89.6]	104/115 (90.4) [83.5-95.1]	.41
CR & BG	48/106 (45.3) [35.6-55.3]	86/223 (38.6) [32.1-45.3	43/115 (37.4) [28.6-46.9]	.42
CR alone	33/106 (31.1) [22.5-40.9]	59/223 (26.5) [20.8-32.8]	33/115 (28.7) [20.7-37.9]	.67
BG alone	11/106 (10.4) [5.3-17.8]	45/223 (20.2) [15.1-26.1]	28/115 (24.4) [16.8-33.2]	**.02**
CR (any)	81/106 (76.4) [67.2-84.1]	145/223 (65.0) [58.4-71.3]	76/115 (66.1) [56.7-74.7]	.94
BG (any)	59/106 (55.7) [45.7-65.3]	131/223 (58.7) [52.0-65.3]	71/115 (61.7) [52.2-70.7]	.66
14-3-3	90/107 (84.1) [75.8-90.5]	175/226 (77.4) [71.4-82.7]	53/108 (49.1) [39.3-58.9]	**<.001**
RT-QuIC	59/68 (86.8) [76.4-93.8]	126/131 (96.2) [91.3-98.7]	59/67 (88.1) [77.8-94.7]	**.03**

Sensitivity defined the percentage of cases with a positive outcome on an investigation or for overall classification as probable sCJD. P values from X^2 test

Groups defined by quartiles: short, 1st quartile; typical, quartiles 2-3; prolonged, 4th quartile

Abbreviations: BG, basal ganglia. CR, cortical ribboning. EEG, electroencephalography. MRI, magnetic resonance imaging. RT-quIC, real-time quaking induced conversion.

© 2022 Watson N et al. *JAMA Network Open.*

eTable 5. Criteria and investigation sensitivity, sCJD cases stratified by age

	Age (years)				
	**<50 (n=19)**	**50-59.9 (n=70)**	**60-69.9 (n=170)**	**70-79.9 (n=168)**	**≥80 (n=70)**
Sensitivity (%) [95% CI]					
Diagnostic criteria					
Probable sCJD					
Any investigation					
Revised	18/19 (94.7) [74.0-99.9]	62/69 (89.9) [80.2-95.8]	152/164 (92.7) [87.6-96.2]	155/164 (94.5) [89.8-97.5]	60/68 (88.2) [78.1-94.8]
Prior	16/19 (84.2) [60.4-96.6]	58/69 (84.1) [73.3-91.8]	122/164 (74.4) [67.0-80.9]	131/164 (79.9) [72.8-85.7]	49/68 (72.1) [59.9-82.3]
All investigations					
Revised	11/11 (100.0) [71.5-100.0]	29/31 (93.6) [78.6-99.2]	74/74 (100.0) [95.1-100.0]	74/75 (98.7) [92.8-100.0]	28/30 (93.3) [77.9-99.2]
Prior	9/11 (81.8) [48.2-97.7]	26/31 (83.9) [66.3-94.6]	55/74 (74.3) [62.8-83.8]	59/75 (78.6) [67.7-87.3]	20/30 (66.6) [47.2-82.7]
Investigations					
EEG	6/17 (35.3) [14.2-61.7]	24/62 (38.7) [26.6-51.9]	77/156 (49.4) [41.3-56.5]	69/147 (46.9) [38.7-55.3]	30/63 (47.6) [34.9-60.6]
MRI (all)	18/19 (94.7) [74.0-99.9]	63/67 (94.0) [85.4-98.3]	134/152 (88.2) [81.9-92.8]	129/150 (86.0) [79.4-91.1]	48/63 (76.2) [63.8-86.0]
CR & BG	8/19 (42.1) [20.3-66.5]	37/67 (55.2) [42.6-67.4]	64/152 (42.1) [34.2-50.4]	58/150 (38.7) [30.8-47.0]	13/63 (20.6) [11.5-32.7]
CR alone	2/19 (10.5) [1.3-33.1]	9/67 (13.4) [6.3-24.0]	44/152 (29.0) [21.9-36.9]	50/150 (33.3) [25.9-41.5]	22/63 (34.9) [23.3-48.0]
BG alone	8/19 (42.1) [20.3-66.5]	17/67 (25.4) [15.5-37.5]	26/152 (17.1) [11.5-24.1]	21/150 (14.0) [8.9-20.6]	13/63 (20.6) [11.5-32.7]
CR (any)	10/19 (52.6) [28.9-75.6]	46/67 (68.7) [56.2-79.4]	108/152 (71.1) [63.2-78.1]	108/150 (72.0) [64.1-79.1]	35/63 (55.6) [42.5-68.1]
BG (any)	16/19 (84.2) [60.4-96.6]	54/67 (80.6) [69.1-89.2]	90/152 (59.2) [51.0-67.1]	79/150 (52.7) [44.4-60.9]	26/63 (41.3) [29.0-43.4]
14-3-3	15/19 (79.0) [54.4-94.0]	47/67 (70.2) [57.7-80.7]	108/158 (68.4) [60.5-75.5]	107/148 (72.3) [64.4-79.3]	46/57 (80.7) [68.1-90.0]
RT-QuIC	11/13 (84.6) [54.6-98.1]	33/36 (91.7) [77.5-98.3]	82/90 (91.1) [83.2-96.1]	90/96 (93.8) [86.9-97.7]	33/36 (91.7) [77.5-98.3]

Sensitivity defined as positive outcome/total for investigations or for criteria definition as *probable* sCJD.

Abbreviations. BG, basal ganglia. CR, cortical ribboning. EEG, electroencephalography. MRI, magnetic resonance imaging. RT-QuIC, real-time quaking-induced conversion.

© 2022 Watson N et al. *JAMA Network Open.*
